# Supplementary material for: Time-Course Transcriptome Analysis of Aquilegia vulgaris Root Reveals the Cell Wall’s Roles in Salinity Tolerance
Source: Int J Mol Sci. 2023 Nov 17;24(22):16450. doi: 10.3390/ijms242216450 (PMC10671252; doi:10.3390/ijms242216450)
Supplement: Supplementary file 1 [file ijms-24-16450-s001.zip › Figure S1.pdf]

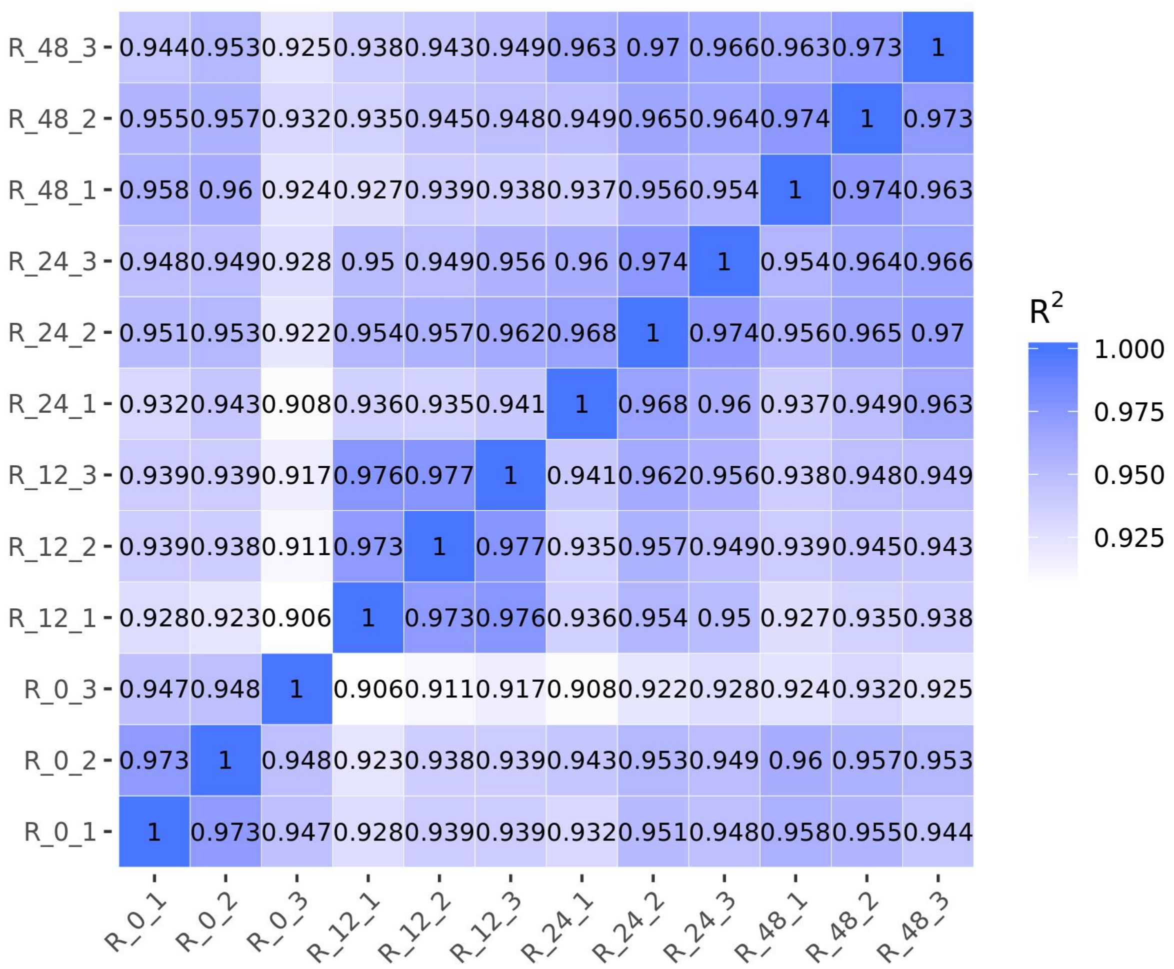

Figure S1. Pearson correlation between samples. The horizontal and vertical coordinates on the graph represent the squared correlation coefficients for each sample.
